# Integrative Systems Biology Analysis Elucidates Mastitis Disease Underlying Functional Modules in Dairy Cattle

**DOI:** 10.3389/fgene.2021.712306

**Published:** 2021-10-08

**Authors:** Nooshin Ghahramani, Jalil Shodja, Seyed Abbas Rafat, Bahman Panahi, Karim Hasanpur

**Affiliations:** ^1^Department of Animal Science, Faculty of Agriculture, University of Tabriz, Tabriz, Iran; ^2^Department of Genomics, Branch for Northwest & West Region, Agricultural Biotechnology Research Institute of Iran (ABRII), Agricultural Research, Education and Extension Organization (AREEO), Tabriz, Iran

**Keywords:** attribute weighting, *E. coli*, machine learning, mastitis, meta-analysis, WGCNA

## Abstract

**Background:** Mastitis is the most prevalent disease in dairy cattle and one of the most significant bovine pathologies affecting milk production, animal health, and reproduction. In addition, mastitis is the most common, expensive, and contagious infection in the dairy industry.

**Methods:** A meta-analysis of microarray and RNA-seq data was conducted to identify candidate genes and functional modules associated with mastitis disease. The results were then applied to systems biology analysis *via* weighted gene coexpression network analysis (WGCNA), Gene Ontology, enrichment analysis for the Kyoto Encyclopedia of Genes and Genomes (KEGG), and modeling using machine-learning algorithms.

**Results:** Microarray and RNA-seq datasets were generated for 2,089 and 2,794 meta-genes, respectively. Between microarray and RNA-seq datasets, a total of 360 meta-genes were found that were significantly enriched as “peroxisome,” “NOD-like receptor signaling pathway,” “IL-17 signaling pathway,” and “TNF signaling pathway” KEGG pathways. The turquoise module (*n* = 214 genes) and the brown module (*n* = 57 genes) were identified as critical functional modules associated with mastitis through WGCNA. *PRDX5, RAB5C, ACTN4, SLC25A16, MAPK6, CD53, NCKAP1L, ARHGEF2, COL9A1*, and *PTPRC* genes were detected as hub genes in identified functional modules. Finally, using attribute weighting and machine-learning methods, hub genes that are sufficiently informative in *Escherichia coli* mastitis were used to optimize predictive models. The constructed model proposed the optimal approach for the meta-genes and validated several high-ranked genes as biomarkers for *E. coli* mastitis using the decision tree (DT) method.

**Conclusion:** The candidate genes and pathways proposed in this study may shed new light on the underlying molecular mechanisms of mastitis disease and suggest new approaches for diagnosing and treating *E. coli* mastitis in dairy cattle.

## Background

Mastitis is a severe disease characterized as an inflammatory condition affecting the mammary glands (Gelasakis et al., 2015). *Escherichia coli, Staphylococcus aureus*, and *Streptococcus uberis* are the three major bacterial pathogens associated with mastitis disease (Vasudevan et al., 2003), with *E. coli* causing severe inflammation in dairy cattle (Vangroenweghe et al., 2005). The focus of current research has shifted to elucidating the underlying mechanisms and developing effective treatment strategies for mastitis disease (Takeshima et al., 2008; Compton et al., 2009). *E. coli* typically infects the mammary glands during the dry period, and inflammation progresses during the early stages of lactation (Burvenich et al., 2003). Recent research indicates that *E. coli's* pathogenicity is entirely dependent on a protein called FecA (Blum et al., 2018).

Recent advancements in high-throughput transcriptome profiling technologies, such as microarray and RNA sequencing (RNA-seq), have enabled opportunities for precision critical care medicine to understand better the molecular mechanisms underlying diverse biological functions (Bansal and Di Bernardo, 2007; Farhadian et al., 2020; Panahi et al., 2020). On the other hand, identifying disease biomarkers can aid breeders in optimizing their genetic programs for dairy animals (Kulkarni and Kaliwal, 2013; Duarte et al., 2015; Lai et al., 2017). Previous research identified *TNF*- and *SAA3* (Swanson et al., 2009), *STAT3, MAPK14, TNF* (Gorji et al., 2019), *IL8RB, CXCL6, MMP9* (Li et al., 2019), *IRF9, CCL* (Buitenhuis et al., 2011), *S100A12, MT2A, SOD2* (Mitterhuemer et al., 2010), *CXCL8, CXCL2*, S100A9 (Sharifi et al., 2018), *PSMA6, HCK*, and *STAT1* (Bakhtiarizadeh et al., 2020) as potential biomarkers for mastitis disease.

Meta-analysis is a systematic and quantitative study methodology used to evaluate prior research and reach a conclusion (Haidich, 2010). On the other hand, independent studies have limitations in sample size, statistical power, and the reliability of the results (Panahi and Hejazi, 2021). Meta-analysis has demonstrated that combining *p* values resolves several issues (Rhodes et al., 2002; Tseng et al., 2012; Panahi et al., 2019a). When combining *p* values using Fisher's technique, the null hypothesis is that the actual effect is zero in each of the combined datasets (Evangelou and Ioannidis, 2013), suggesting that the techniques should be sensitive even when only a subset of the combined datasets has an impact magnitude more significant than zero. Fisher's approach outperformed other methods for establishing associations. In addition, the *p* value combination method shows considerable promise for identifying novel markers or differentially expressed genes (DEGs) (Evangelou and Ioannidis, 2013). Moreover, connectivity analysis of known meta-genes has been presented as a promising approach for disentangling the complicated method (Panahi et al., 2020).

Weighted gene coexpression network analysis (WGCNA) has been proposed as a versatile tool for gene coexpression analysis, which provide valuable information about gene connectivity based on gene expression levels (Ebrahimie et al., 2014; Farhadian et al., 2021). A combination of machine-learning algorithms and microarray meta-analysis was used to identify mastitis genes in dairy cattle (Sharifi et al., 2018), However, they did not include RNA-seq data in their analysis and instead focused on the expression patterns of meta-genes.

The present study is the first that the authors are aware of that integrates meta-analysis of microarray and RNA-seq datasets, connectivity analysis, and model optimization in mastitis disease. Thus, in this integrative study, we identified master genes associated with mastitis disease using a combination of meta-analysis, WGCNA, and machine-learning algorithms.

## Materials and Methods

### Data Collection

The National Center for Biotechnology Information's Gene Expression Omnibus (GEO) repository (https://www.ncbi.nlm.nih.gov/gds/) was explored for datasets related to dairy cattle mastitis. This database was searched for RNA-seq and microarray studies using the keywords “*Bos taurus*,” “mastitis,” and “*Escherichia coli*.” For this research, six microarrays and two RNA-seq datasets were chosen. Table 1 lists the platform, accession number, species, and references for each dataset. All healthy and mastitis animal samples were from the *Bos taurus* species, which have a high sensitivity to *E. coli*. Fifteen healthy German Holstein Frisian cows in midlactation (3–6 months postpartum) were included in dataset GSE15025. The animals were inoculated with *E. coli* in one-quarter and died after 6 h (*n* = 5) or 24 h (*n* = 5) in two distinct infection scenarios. Five heifers were used as controls; they were not given any medication and died after 24 h. At 4 to 6 weeks following parturition, 16 healthy primiparous Danish Holstein-Friesian cows were tested with *E. coli* for the GSE24217 dataset. The overall udder health of 24 dairy cows was assessed before the disease challenge. Control quarters were selected based on bacteriological tests performed before *E. coli* inoculation and the quarter foremilk SCC at 24 and 192 h. From the total German Holstein population, 11 heifers at day 42 postpartum, either with high or low sensitivity to mastitis, were chosen for the GSE24560 dataset. Heat-inactivated *E. coli* plus *S. aureus* was used to challenge the cells, as a control. The cells were collected after 1, 6, and 24 h, and mRNA expression was compared. Four first-lactation Holstein cows in the fourth month of lactation were also experimentally inoculated with the mastitis-causing *E. coli* pathogen strain 1303 for the GSE25413 dataset. The transcriptomes of the treated and untreated cells were examined at 1, 3, 6, and 24 h. In the GSE32186 dataset, four first-lactation Holstein cows were given primary MEC (pbMEC) cultures for 6 h, and some cultures were stimulated. *E. coli* particles were collected from the udders of three healthy, pregnant (day 130 of gestation) cows in the middle of their first lactation 12 or 42 h later. Six Holstein Friesian cows were challenged with *E. coli* mastitis for the GSE50685 investigations. Every 6 h after infection, blood and milk samples were taken. At successive milkings, the treatment was repeated (12, 24, and 36 h postchallenge). At 24 h (*n* = 3) and 48 h (*n* = 3) following infection, the cows were sacrificed for tissue collection. GSE75379 and GSE159286 were two datasets related to RNA-seq. Sixteen healthy primiparous Holstein cows at 4–6 weeks of lactation were included in the GSE75379 dataset. Biopsy specimens of healthy and diseased udder tissue were taken at *T* = 24 h and *T* = 192 h after infection. In total, 12 heifers were intramuscularly vaccinated with heat-killed *E. coli* for the GSE159286 investigations. Half of the heifers (IM group, n = 6) received a booster injection 2 months later. Others (IMM group, n = 6) received 50 g of protein concentrate produced from *E. coli* in two quarters. Cows were then challenged with an *E. coli* P4 bacterial suspension infusion at 49 days in milk inside one healthy quarter (10e3 bacteria). Before the trial, blood was taken 7 days after the first and second injections (immunization phase) and then at 0, 12, and 40 h following infection (challenge phase).

**Table 1:** Selected microarray and RNA-seq datasets for systems biology analysis of mastitis disease.

**Accession no**.	**Species**	**Bacteria**	**Platform**	Samples^*^ (C:T)	**References**
**Microarray datasets**					
GSE15025	*Bos taurus*	*Escherichia coli*	Affymetrix	15:15	Mitterhuemer et al., 2010
GSE24217	*B. taurus*	*E. coli*	Affymetrix	23:26	Buitenhuis et al., 2011
GSE24560	*B. taurus*	*E. coli*	Affymetrix	27:31	Brand et al., 2011
GSE25413	*B. taurus*	*E. coli*	Affymetrix	6:24	Günther et al., 2011
GSE32186	*B. taurus*	*E. coli*	Affymetrix	12:12	Günther et al., 2012
GSE50685	*B. taurus*	*E. coli*	Affymetrix	5:15	Sipka et al., 2014
**RNA-seq datasets**					
GSE75379	*B. taurus*	*E. coli*	Illumina	6:12	Moyes et al., 2016
GSE159286	*B. taurus*	*E. coli*	Illumina	53:52	Cebron et al., 2020

* *Number of healthy and infected samples*.

### Preprocessing and Analysis of Microarray Datasets

The GEO database was used to collect all microarray expression raw data and associated annotations for each study, and microarray data were preprocessed to obtain reliable findings. Nonbiological data variances were then removed, and appropriate scales were used for further analysis (Bolstad et al., 2003). The quantile normalization method and batch effects reduction were used to conduct effective gene expression analysis and eliminate variability between studies. The Limma software (2.16.0) (Smyth et al., 2005) was used to calculate DEGs among each control and treatment group after preprocessing the raw data. DEGs were deemed significant when the false discovery rate (FDR) using the Benjamini–Hochberg method was *p* < 0.05 and the logarithm of fold change > ±0.5 (Benjamini and Hochberg, 1995).

### Preprocessing and Analysis of Individual RNA-seq Datasets

The data generated by RNA-seq can be skewed due to biases introduced during library preparation, polymerase chain reaction, and sequencing. The technique of trimmed mean of m-values was used to eliminate the effect of known nonbiological features on the RNA-seq data (Robinson and Oshlack, 2010). Each sample was inspected for quality using the FastQC tool version 0.11.5 (Trapnell et al., 2012), and low-quality reads were trimmed using the Trimmomatic (v 0.32) software (Goldman et al., 2006). Bowtie2 (2.2.4) software was used to index reference genomes, and clean reads were then mapped to the *B. taurus* reference genome (ARS-UCD1.2 version) employing Tophat2 (2.0.10) software (Kim et al., 2013; Love et al., 2014) with default settings. The sample mapping rates are listed in Supplementary Material 1, Table 1. The htseq-count package (2.7.3) (Anders et al., 2013) was used to calculate the expression count matrix. The Bioconductor DESeq2 software (1.10.1) was used to determine the differential gene expressions in each research (Love et al., 2014). In terms of normalization and batch effect correction, the methods outlined in the studies (Farhadian et al., 2021; Panahi and Hejazi, 2021) were followed. In summary, genes with a CV <10% were initially removed. The group was then used as a covariate, using DESeq2 library size, size factor normalization factors. The variance-stabilizing transformations (VSTs) function was used to reduce sample disparities. The VST function does not typically remove variations related to the batch or other variables. As a result, the “removeBatchEffect” function was used to remove batch variations. The blind = false option was selected as re-estimation of the dispersion values was not required. This process leveled the library size and other normalization variables. Each study's samples were normalized jointly, implying that each dataset was normalized individually (Love et al., 2014).

### Meta-Analysis of Microarray and RNA-seq Datasets

**Figure 1:**
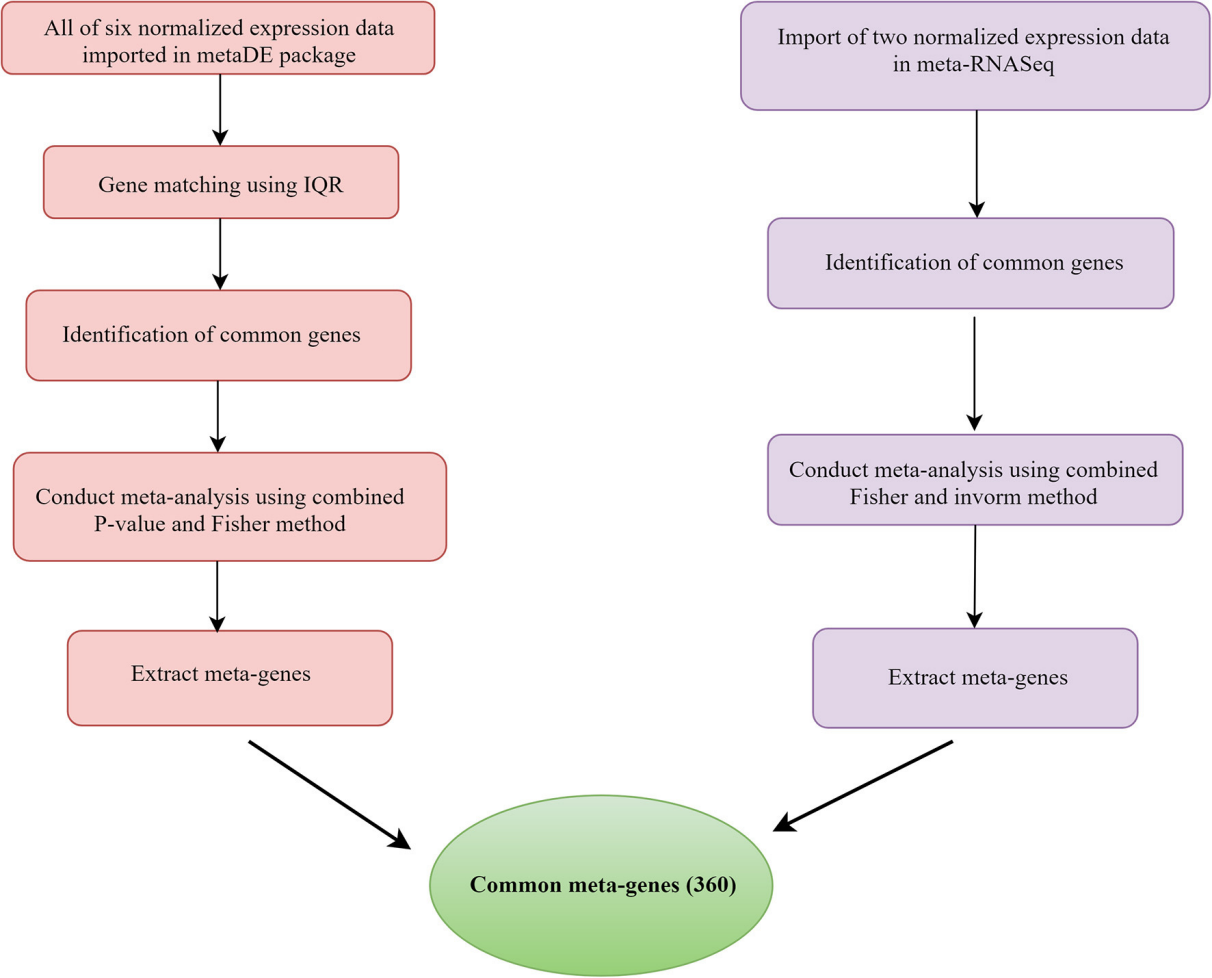
Flowchart of different steps of mastitis microarray and RNA-seq meta-analysis based on combing *p* value.

In microarray studies, the MetaDE package (1.0.5) was used to identify meta-genes (Wang et al., 2012). The meta-analysis included the following steps: after quantile normalization, labeling samples as “Infected” or “Healthy”; the “Gene Symbol” was matched to multiple probe IDs using the interquartile range for probe selection (Wang et al., 2012). Merging genes is an approach used to determine which genes should be studied further (Wang et al., 2021). Because the number of genes in the research varied, the multiple gene expression datasets may not have been adequately matched by genes. In this study, the direct merging method was used to obtain common genes across different investigations. The Fisher technique (Marot et al., 2020) was used to identify meta-gens in RNA-seq data using the metaRNASeq software (1.0.5). Initially, the DEGs for each study were defined using the DESeq2 package (1.30.1), and the corresponding *p* value was extracted. Then, the fishcomb function included in the metaRNASeq package was used to combine the *p* values. For downstream analysis, the genes shared between meta-analyses of microarray and RNA-seq data were extracted (Figure 1).

### Weighted Gene Coexpression Network Analysis

Common genes were used to construct coexpression networks using the WGCNA Bioconductor R package (version 3.5.1) to understand the correlation patterns among genes and identify significant modules associated with mastitis disease (Langfelder and Horvath, 2008). Initially, networks were constructed using Pearson correlations across all common genes (Botía et al., 2017). A soft threshold was used to evaluate the correlation and noise-filtering power to fulfill the scale-free topology requirement. The weighted gene coexpression network design promotes strong correlations at the cost of low correlations by increasing the absolute magnitude of the correlation to a soft threshold (Langfelder and Horvath, 2008). The topological overlap matrix (TOM) and its corresponding dissimilarity matrix (1–TOM) were used to visualize the network, which resulted in a network diagram for module detection. Module eigengenes and module membership were used to identify the hub genes for each significant coexpressed module (Langfelder and Horvath, 2008). The following parameters were used to construct the modules: cut height of 0.975, minimum module size of 30 genes, “hybrid” method, and deepSplit = 2.

### Functional Enrichment Analysis

The STRING (Szklarczyk et al., 2015) database was used to conduct enrichment analysis on the Kyoto Encyclopedia of Genes and Genomes (KEGG) and Gene Ontology (GO) (Dennis et al., 2003). The FDR (<0.05) correction was used to determine the statistical significance of GO and KEGG terms.

### Protein–Protein Interaction Networks of Common Genes

Gene network analysis of protein–protein interaction between common genes was performed using Cytoscape software to visualize gene networks and identify hub genes. Hub genes are defined as those with the highest degree of connectivity and those with a greater biological significance than other gene members (Shannon et al., 2003).

### Supervised Machine-Learning Models

**Figure 2:**
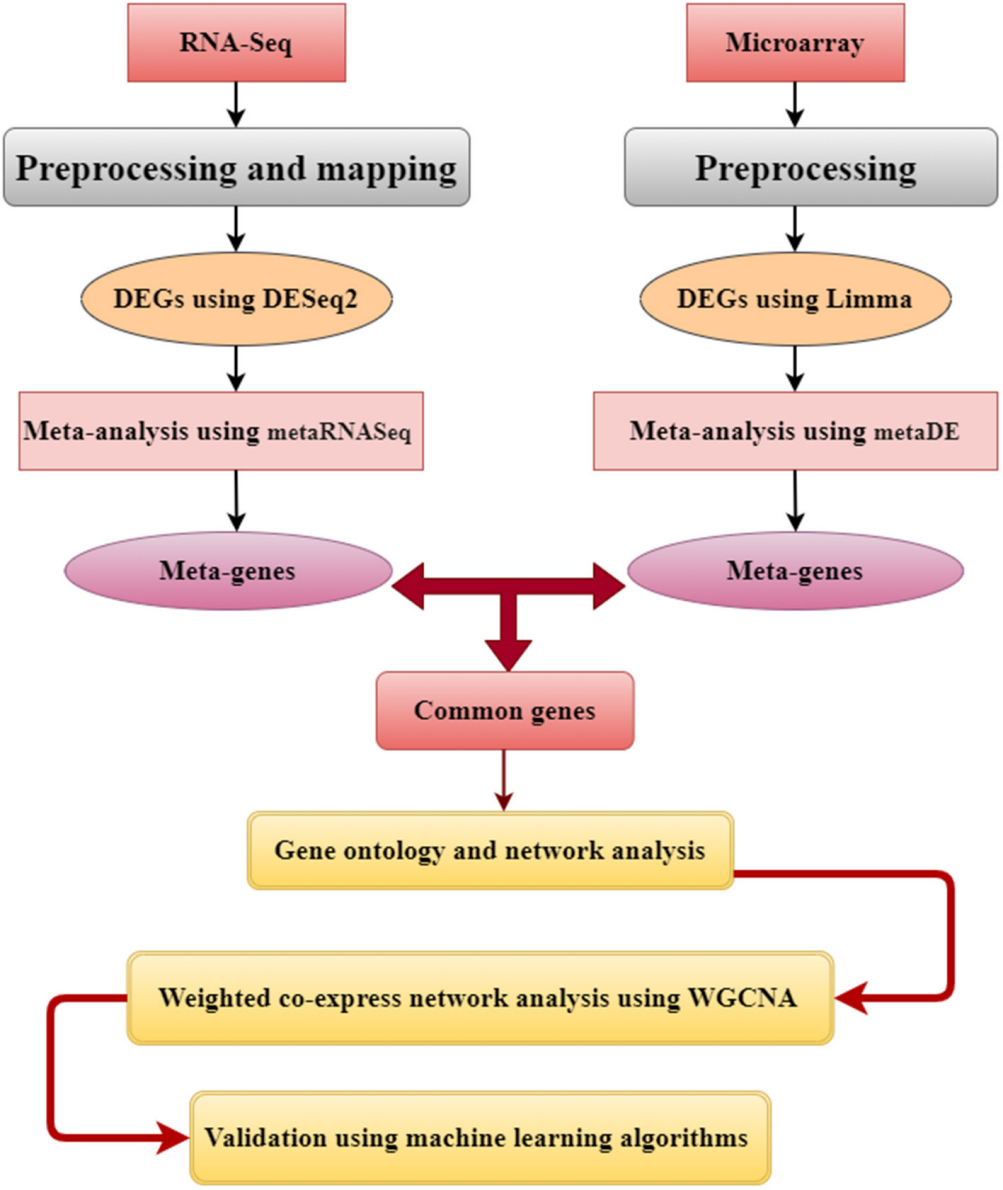
Flowchart of applied systems biology approach in this study.

The common meta-genes identified were utilized to select features using 10 different weighting algorithms, including information gain, information gain ratio, χ^2^, deviation, rule, support vector machine, Gini index, uncertainty, relief, and PCA to validate the hub genes' efficacy in distinguishing different genes involved in mastitis disease (Farhadian et al., 2018a, 2021). The Rapid Miner software (Rapid Miner 5.0.001, Dortmund, Germany) was used for attribute weighting (Ebrahimi et al., 2011; Farhadian et al., 2018a; Panahi et al., 2019a; Nami et al., 2021). The primary objective of attribute weighting algorithms was to extract a subset of input features (genes) by excluding those that contained little or no information (Panahi et al., 2019b). The decision trees (DTs) were constructed using features with weighting values greater than 0.5. The DTs were constructed using the following methods: information gain, information gain ratio, Gini index, and accuracy criteria. Figure 2 depicts the flowchart of an analytical strategy for microarray and RNA-seq.

## Results

### Meta-Analysis

**Figure 3:**
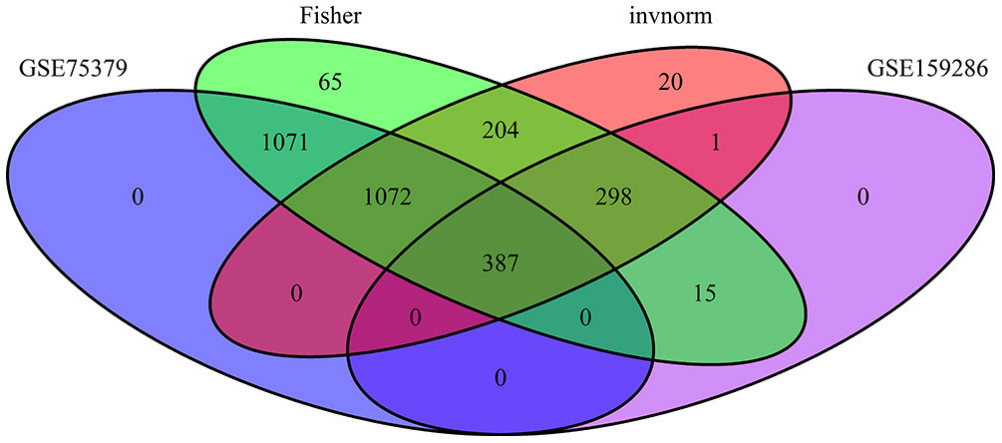
Results of meta-analysis of RNA-seq data using Fisher and invorm method.

We conducted a meta-analysis of DEGs using data from microarray and RNA-seq experiments. Six raw microarray datasets containing 211 samples and two RNA-seq datasets containing 123 independent dairy cattle experiments were chosen separately for the meta-analysis. Finally, a total of 2,089 and 2,794 meta-genes in response to *E. coli* mastitis in microarray and RNA-seq data, respectively, were observed using the Fisher method in the metaDE and metaRNASeq packages. Supplementary Material 2 and Figure 3 contain the results of the meta-analysis of RNA-seq data.

### Identification of Common Genes by Meta-Gene Comparison

**Figure 4:**
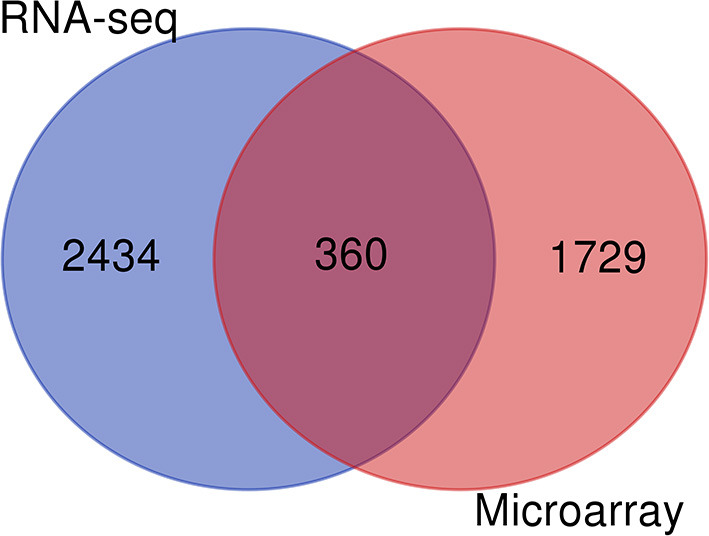
Results of common meta-genes using Venn diagram.

A total of 360 genes were identified as common meta-genes in meta-analysis of microarray and RNA-seq data (Figure 4). Table 2 and Supplementary Material 3 contain additional information about the significant common meta-genes.

**Table 2:** Significant common meta-genes in mastitis disease.

**Gene symbol**	**Full name**	**Chromosome**	**Compartment**
*ELMO2*	Engulfment and cell motility 2	20	Cytosol
*ORM1*	Orosomucoid 1 (alpha-1-acid glycoprotein)	9	Extracellular
*ABCB7*	ATP-binding cassette subfamily B member 7	X	Mitochondrion
*LRRC41*	Leucine-rich repeat containing 41	1	Nucleus
*CXCL3*	C-X-C motif chemokine ligand 3	4	Extracellular
*SOD2*	Superoxide dismutase 2	6	Extracellular
*ZFYVE1*	Zinc finger FYVE-type containing 1	14	Mitochondrion
*SORL1*	Sortilin-related receptor 1	11	Plasma membrane
*PTCD3*	Pentatricopeptide repeat domain 3	2	Mitochondrion
*RPS6KA5*	Ribosomal porotein S6 kinase A5	14	Nucleus
*LHFPL2*	LHFPL tetraspan subfamily member 2	5	Plasma membrane
*TRIQK*	Triple QxxK/R motif containing	8	Endoplasmic reticulum
*MAOA*	Monoamine oxidase A	X	Mitochondrion
*CORO2A*	Coronin 2A	9	Cytosol
*TPM3*	Tropomyosin 3	1	Extracellular
*PTPRC*	Protein tyrosine phosphatase receptor type C	1	Plasma membrane
*LOC407171*	Fc gamma 2 receptor	18	Extracellular

### Functional Enrichment Analysis of Common Genes

The STRING database was used to conduct GO analyses on 360 common meta-genes to ascertain their biological process (BP), molecular function (MF), and cellular component (CC) roles in mastitis disease. The results found 170, 33, and 36 GO terms for BPs, MFs, and CCs, respectively. The terms “cellular process,” “response to stimulus,” “biological regulation,” “regulation of a biological process,” and “regulation of a cellular process” were used to denote the most critical process in the BP category. “Binding,” “ion binding,” “actin binding,” “cation binding,” and “metal ion binding” were all significantly overrepresented in the MF category. In terms of CC, the terms “intracellular,” “cell,” “cytoplasm,” “intracellular organelle,” and “organelle” were significantly enriched. Additional information is available in Table 3 and Supplementary Material 4.

**Table 3:** Significant GO terms of common genes.

**Term ID**	**Description**	**GO terms**	***p*** **value**
**GO:0009987**	Cellular process	BP	1.52E-10
**GO:0050896**	Response to stimulus	BP	1.96E-10
**GO:0065007**	Biological regulation	BP	8.53E-10
**GO:0050789**	regulation of BP	BP	3.66E-09
**GO:0050794**	Regulation of cellular process	BP	3.66E-09
**GO:0051716**	Cellular response to stimulus	BP	6.09E-08
**GO:0019222**	Regulation of metabolic process	BP	2.26E-07
**GO:0051171**	Regulation of nitrogen compound metabolic process	BP	2.26E-07
**GO:0080090**	Regulation of primary metabolic process	BP	2.26E-07
**GO:0031323**	Regulation of cellular metabolic process	BP	2.63E-07
**GO:0005488**	Binding	MF	3.90E-10
**GO:0043167**	Ion binding	MF	2.55E-06
**GO:0005515**	Protein binding	MF	1.30E-05
**GO:0003779**	Actin binding	MF	0.00011
**GO:0043169**	Cation binding	MF	0.00024
**GO:0046872**	Metal ion binding	MF	0.00029
**GO:0008092**	Cytoskeletal protein binding	MF	0.00034
**GO:1901363**	Heterocyclic compound binding	MF	0.0014
**GO:0097159**	Organic cyclic compound binding	MF	0.0019
**GO:0036094**	Small molecule binding	MF	0.002
**GO:0005622**	Intracellular	CC	7.27E-13
**GO:0005623**	Cell	CC	8.74E-13
**GO:0005737**	Cytoplasm	CC	8.74E-13
**GO:0043229**	Intracellular organelle	CC	1.39E-09
**GO:0043226**	Organelle	CC	1.74E-09
**GO:0043227**	Membrane-bound organelle	CC	5.37E-09
**GO:0043231**	Intracellular membrane-bound organelle	CC	7.48E-09
**GO:0005829**	Cytosol	CC	2.03E-07
**GO:0070013**	Intracellular organelle lumen	CC	1.28E-05
**GO:0005634**	Nucleus	CC	3.30E-05

*Only the significantly enriched (p < 0.05) GO terms are presented*.

This analysis identified a total of nine significant KEGG pathways. In addition, the results indicated that the “peroxisome,” “NOD-like receptor signaling pathway,” “IL-17 signaling pathway,” and “TNF signaling pathway” were significantly overrepresented. Table 4 contains additional information about KEGG pathways.

**Table 4:** The significant KEGG metabolic pathways associated with the common genes.

**Pathway name**	***p*** **value**	**Total genes in pathway**	**Strength**
Peroxisome	0.0012	10	0.84
NOD-like receptor signaling pathway	0.0058	12	0.61
IL-17 signaling pathway	0.0058	9	0.76
TNF signaling pathway	0.008	9	0.68
Salmonella infection	0.008	8	0.76
Viral carcinogenesis	0.008	13	0.55
Human papillomavirus infection	0.0089	16	0.46
Necroptosis	0.0194	10	0.56
Autophagy—animal	0.0216	9	0.59

*TNF, tumor necrosis factor*.

**Figure 5:**
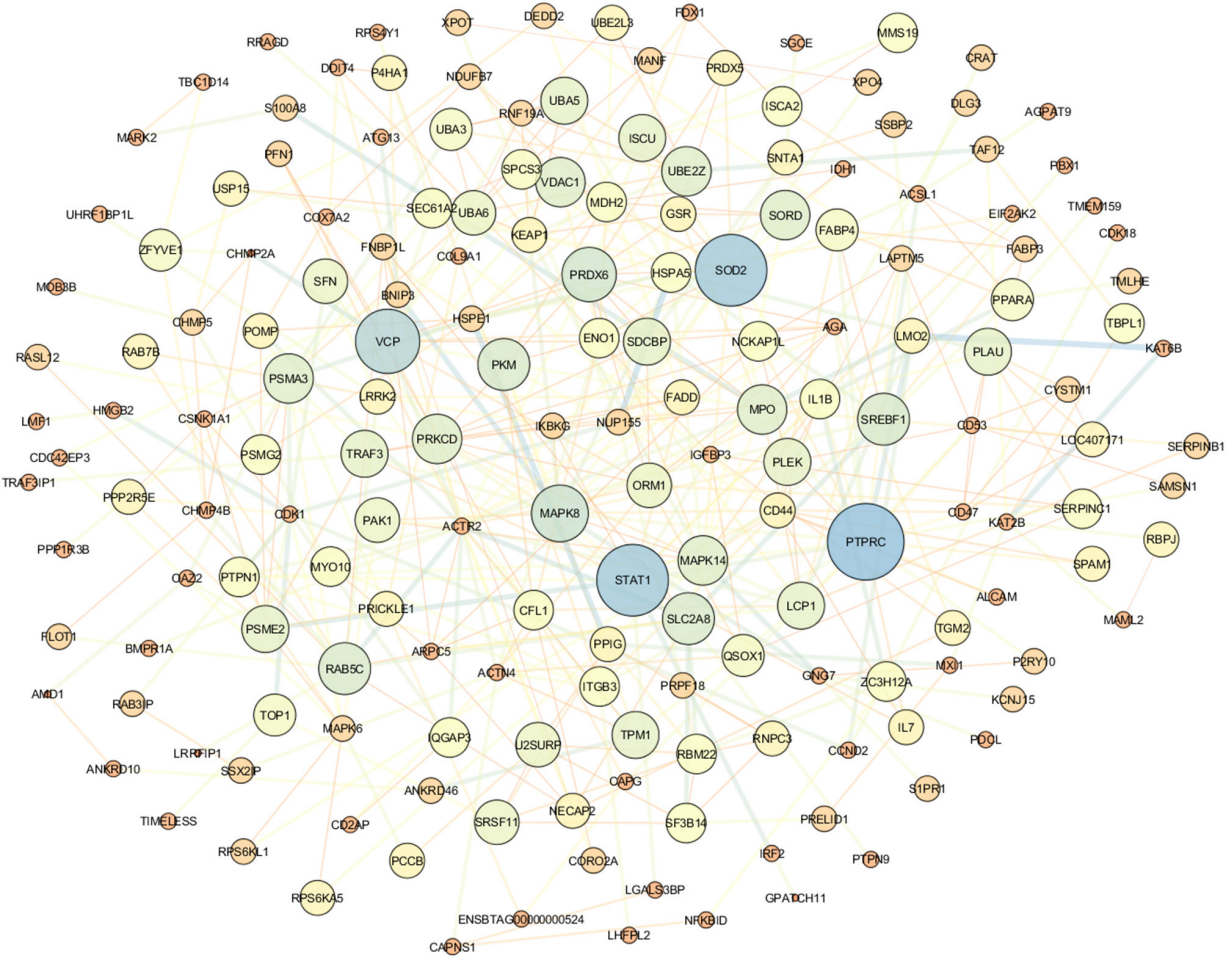
Protein–protein interaction network for the common genes using Cytoscape.

Cytoscape demonstrates the involvement of DEGs in protein–protein interaction. Figure 5 illustrates the gene network visualization of common meta-genes. The top genes were STAT1, RTPRC, SOD2, and VCP (Supplementary Material 5).

### Weighted Gene Coexpression Network Construction

**Figure 6:**
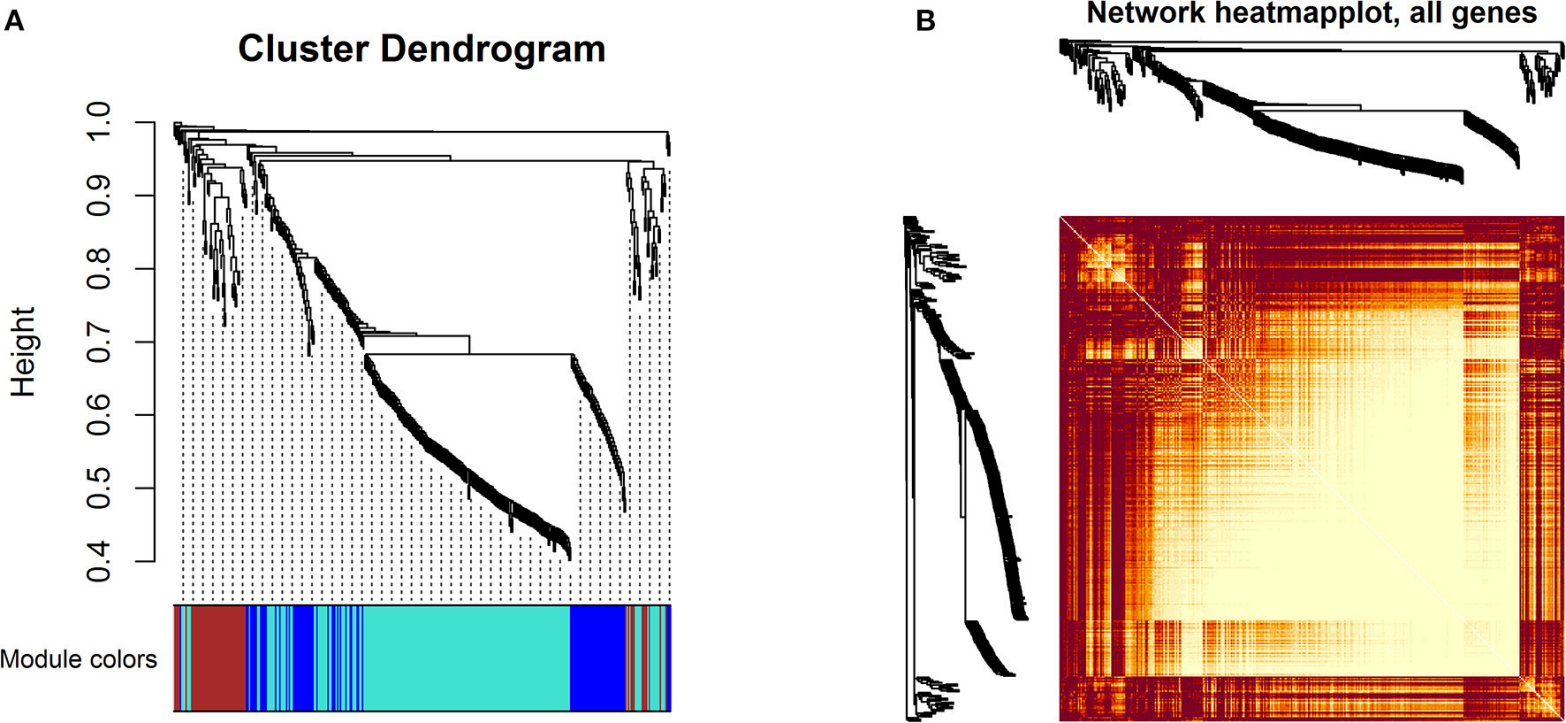
WGCNA: **(A)** Cluster dendrogram of the common genes. The branches and color bands demonstrate the specific module. **(B)** TOM plot; light color symbolizes low overlap, and progressively darker red color symbolizes higher overlap between common genes. Blocks of darker colors along the diagonal correspond to modules.

A WGCNA was performed to identify genes with a high correlation and classified the common genes into four modules. The turquoise module (*n* = 214 genes) and the brown module (*n* = 57 genes) were identified as critical functional modules associated with mastitis through WGCNA analysis (Figure 6A). The remaining modules, such as the blue module (*n* = 84 genes) plus the gray module (*n* = 5 genes), were not notable. Figure 6B illustrates the hierarchical clustering of common genes.

**Figure 7:**
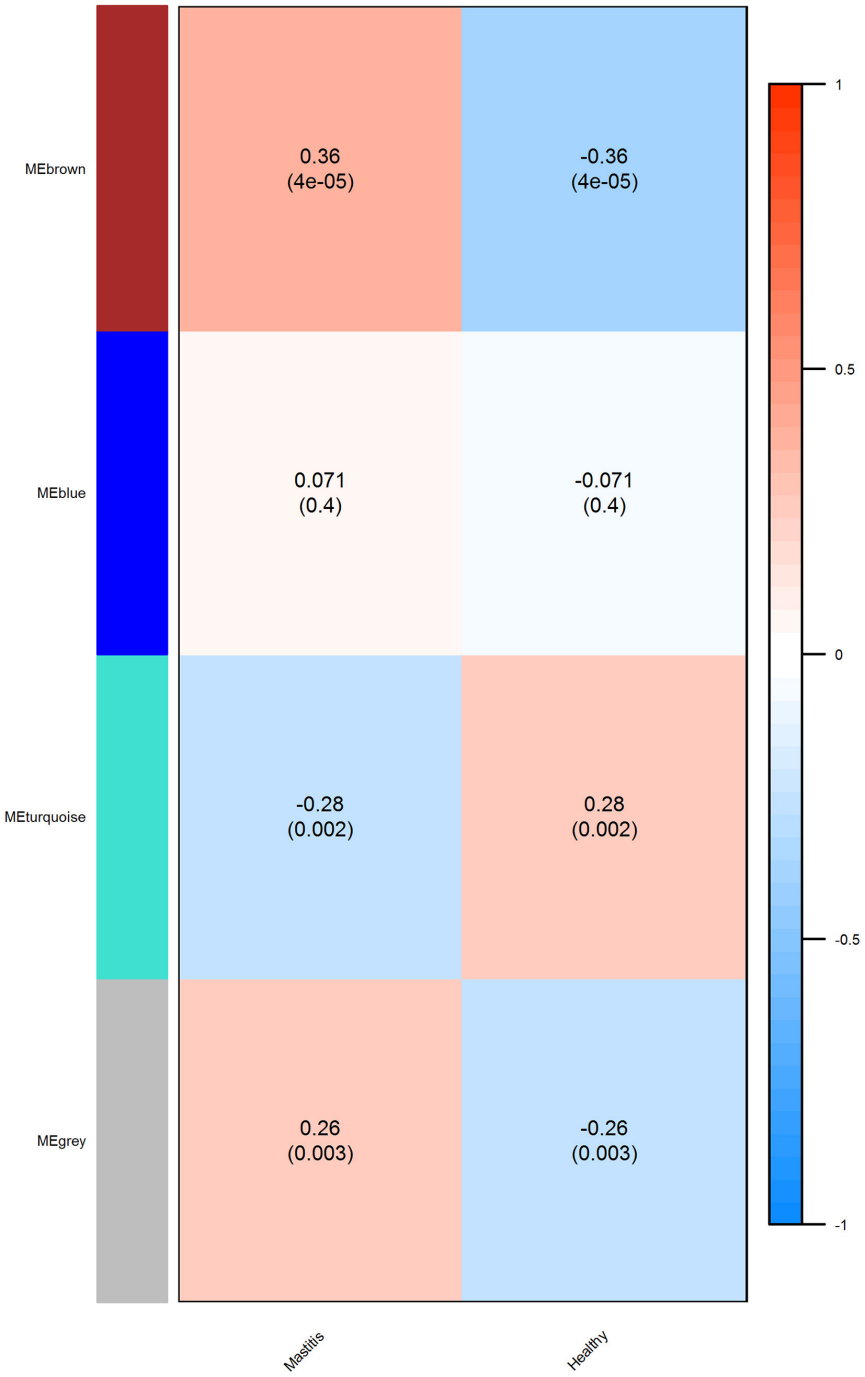
The module trait relationship (*p* value) for identified modules (y axis) in relation with traits (x axis). The relationship was colored according to the correlation between the module and traits (turquoise, strong negative correlation; brown, strong positive correlation).

The correlation coefficient and *p* value for the significant modules in the mastitis and healthy groups were *r* = 0.28, *p* = 0.002, and *r* = 0.36, *p* = 4e – 05, respectively, for the turquoise and brown modules (Figure 7).

Turquoise had a negative correlation with mastitis disease, whereas brown had a positive correlation. Table 5 lists the top five hub genes in brown and turquoise modules. Supplementary Material 6 contains a list of the more significant modules identified.

**Table 5:** Top hub genes in significant modules in mastitis disease.

**Mastitis disease**	
**Brown**	**Turquoise**
*PRDX5*	*CD53*
*RAB5C*	*NCKAP1L*
*ACTN4*	*ARHGEF2*
*SLC25A16*	*COL9A1*
*MAPK6*	*PTPRC*

“Peroxisome,” “viral carcinogenesis,” and “arginine and proline metabolism” were determined as the most significantly enriched pathways based on the enriched functional analysis in these modules that were potentially associated with mastitis development. These modules enriched for genes involved in “negative regulation of peptidyl-serine phosphorylation,” “response to stimulus,” “cell process regulation,” “protein hydroxylation,” “actin filament-based process,” and “cellular process.” These modules carried out critical MFs such as “actin binding,” “binding,” “transcription factor binding,” and “peroxidase activity.” “Intracellular,” “organelle,” “cytoplasm,” “cell,” “cortical actin cytoskeleton,” and “microvillus” were identified as CCs.

### Attribute Weighting

The data-cleaning process was used to eliminate redundant and highly correlated (>95%) attributes. Finally, modeling was performed on the 360 genes. If an attribute was assigned a weight >0.5 by a specific attribute weighting algorithm, it was considered essential. Supplementary Material 7 contains the results of 10 different attribute weighting algorithm applications. Table 6 summarizes the number of attribute weighting algorithms that supported the selected DEGs.

**Table 6:** Results of different attribute weighting algorithms confirmed the most important genes.

**Attribute**	**No. of weighting models**
*LOC407171*	5
*MT2A*	4
*PTPRC*	4
*LPCAT2*	4
*SAMSN1*	4
*IL1B*	4
*SELPLG*	4
*CD53*	4
*PLEK*	4
*SFN*	3
*KCNJ15*	3
*SPCS3*	3
*SOD2*	3
*IDH1*	3
*SYNGR1*	3
*TANC2*	3
*CXCL3*	3

### Validation Hub Genes in Coexpressed Modules

The DT technique was used to validate the identified hub genes. Thus, the accuracy of various models was calculated and presented in Supplementary Material 8 using four different criteria, namely, information gain ratio, information gain, Gini index, and accuracy. According to the results, the DT with the gain ratio criterion achieved the highest accuracy (75%) (Table 7). The DT validated the role of the top-ranked genes in mastitis classification using the expression values of common meta-genes.

**Table 7:** Accuracy comparison of constructed DT models by different criteria.

**Criteria**	**Accuracy (%)**
Gain ratio	75
Information gain	63.89
Gini index	58.33
Accuracy	63.89

**Figure 8:**
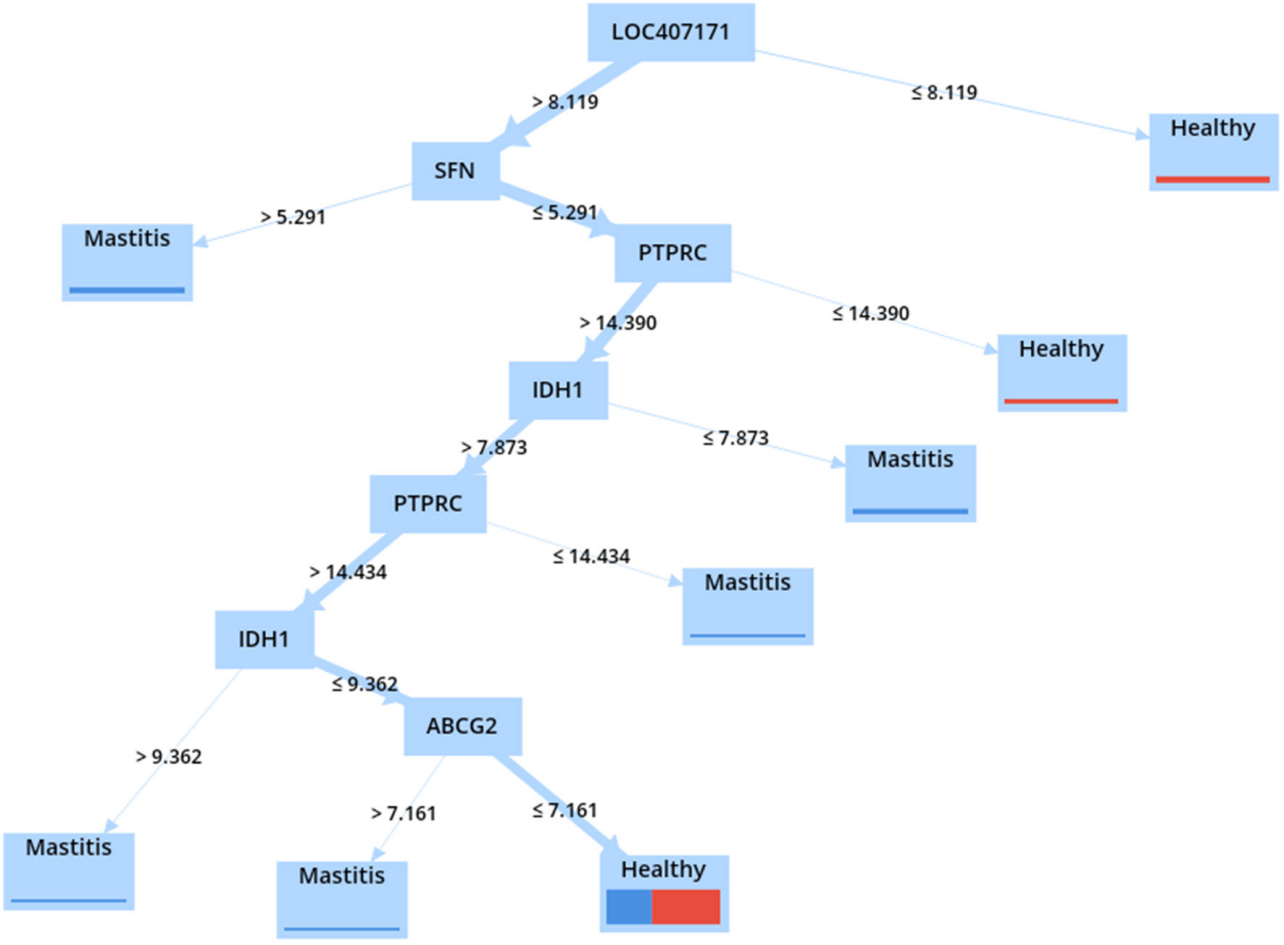
DT model using gain ratio criterion in healthy and mastitis samples.

As illustrated in Figure 8, because the *LOC407171* gene is located at the root of the constructed tree, it can be considered a biomarker for mastitis. When the *LOC407171* gene value exceeded 8.119, and the *SFN* gene value exceeded 5.291, the samples were classified as having mastitis. When the *LOC407171* gene value is equal to or <8.119, the sample is considered healthy. When *LOC407171* exceeded 8.119, SFN was equal to or <5.291, and *PTPRC* was equal to or <14.390, the sample was classified as healthy. In addition, if the last feature exceeded 14.390 and the expression of *IDH1* was present, *PTPRC* would be classified as having mastitis.

The significance of the *LOC407171, PTPRC, ABCG2*, and *IDH1* genes in the turquoise module was confirmed using DT models and attribute weighting, highlighting their critical roles in mastitis.

## Discussion

Mastitis is a significant disease involving multiple genes that may interact to enrich specific signaling pathways. We performed a meta-analysis of RNA-seq and microarray transcriptome data to gain a comprehensive understanding of the master/key genes during mastitis disease that may play a significant role in response to *E. coli* mastitis. As individual studies have limitations in statistical power and reproducibility, several small impact genes remain unknown. Meta-analysis has been suggested as a practical approach for resolving this issue (Farhadian et al., 2018b; Sharifi et al., 2018). The BP, biological regulation, and reaction to a stimulus, the study's primary enriched GO terms, have been described as BPs in mastitis disease (Asselstine et al., 2019). These words include various activities, including cell proliferation, cell growth, biochemical processes, and signaling pathways (Long et al., 2001; Arnellos, 2018). The terminology used to describe MF in this research, such as binding, ion binding, protein binding, actin binding, cation binding, and catalytic activity, has been previously described in immune response and protein transport (Swanson et al., 2009; Asselstine et al., 2019).

Enrichment analysis of metabolic KEGG pathways was used to identify metabolic pathways that were significantly overrepresented among 360 common genes. Several significant pathways were enriched, including peroxisomes and three subcategories of signaling pathways [interleukin 17 (IL-17) signaling pathway, nucleotide-binding and oligomerization domain (NOD)–like receptor signaling pathway, TNF signaling pathway]. Peroxisomes are required to oxidize specific biomolecules and the inflammatory response to environmental stress (Trindade Da Rosa, 2016; Su et al., 2019). Mammary epithelial cells have been shown to have immune activity, activating signaling pathways during mastitis (Song et al., 2014). TNF plays a role in various pathological processes, including immune cell regulation and immune response modulation (Shah et al., 2012; Gao et al., 2015). The NOD-like receptor regulates the immune and inflammatory responses in mammals' innate immune systems (Saxena and Yeretssian, 2014). IL-17 expression in milk peaked 24 to 48 h after pathogen challenge. These findings indicated that IL-17 was a significant cytokine in the development of dairy goat mastitis and played a critical role in mastitis development (Jing et al., 2012). Previously published research indicated that mastitis involves the NOD-like receptor, IL-17, and TNF signaling pathways (Asselstine et al., 2019). As a result of their function, we can deduce that these pathways are involved in immune system responses to mastitis disease.

The PPI networks constructed using Cytoscape revealed that the hub genes are *PTPRC*, SOD2, and *STAT1*. As a result, these hub genes may affect mastitis and thus warrant further validation. The *PTPRC* gene is required to signal T- and B-cell antigen receptors (Miterski et al., 2002; Porcu et al., 2012). *PTPRC* is a highly connected gene in PPI networks and is involved in the development of mastitis (Bakhtiarizadeh et al., 2020). *SOD2* and *IDH1* genes have been up-regulated in ewes' mammary glands using functional enrichment analysis (Gao et al., 2018, 2019). *SOD2* gene expression increased in mammary tissue of cows and ewes with mastitis caused by *S. aureus* and *E. coli* (Mitterhuemer et al., 2010; Jensen et al., 2013). Also, in addition, *STAT1* regulates genes involved in milk protein synthesis, fat metabolism, and immune cell activation (Cobanoglu et al., 2006). The analysis of common meta-gene coexpression networks identified four modules, two of which were significant. These modules were the most significant in the current study based on the enriched functional terms related to mastitis development. The brown module's most essential genes included *PRDX5, RAB5C, ACTN4*, and *MAPK6*. The *PRDX5* gene is expressed ubiquitously in tissues and protects cells from oxidative stress by detoxifying peroxides (Knoops et al., 2011). *PRDX5* has been shown to play a critical role in inflammation in mice by protecting cells from oxidative stress (Argyropoulou et al., 2016). In addition, the *PRDX5* gene expression is increased in mastitis sheep milk (Pisanu et al., 2015). *RAB5C* and *MAPK6* genes were identified as candidate genes for mastitis in dairy cattle following intramammary infection with *E. coli* or *S. uberis* using a combination of GWAS and DEG data analyses (Chen et al., 2015). The ACTN4 gene was identified as the DEG in mastitis vs. healthy samples of sheep milk by transcriptomic analysis (Bonnefont et al., 2011). Furthermore, ACTN4 was identified as a hub gene in mastitis-related modules (Bakhtiarizadeh et al., 2020). On the other hand, the turquoise module's master genes were the *CD53, ARHGEF2*, and *COL9A1* genes. *CD53* regulates cell development, and its function has been implicated in mastitis disease (Rinaldi et al., 2010). The results of a high-throughput analysis on infected bovine mammary glands with *E. coli* indicated the *ARHGEF2* gene's importance (Bagnicka et al., 2021). The *COL9A1* gene has been implicated in research involving identifying genomic regions and expression analysis of mastitis (Lu et al., 2020).

Several genes, including *LOC407171, MT2A, LPCAT2, CXCL3, SFN, IDH1*, and *ABCG2*, were confirmed as essential genes based on the outcome of the attribute weighting algorithm. The *LOC407171* gene is associated with the innate immune response in beef cattle and has been identified as an up-regulated gene in a dairy cow with *E. coli* mastitis (Li et al., 2019). *MT2A* plays a role in stimulus response in the pathogenesis of bovine *E. coli* in early lactation cows (Cheng et al., 2021). *LPCAT2* regulates the glycerophospholipid metabolism in periparturient dairy cattle (Bakhtiarizadeh et al., 2020). *CXCL3* is recognized as a proinflammatory cytokine in dairy cows with experimentally induced *S. aureus* clinical mastitis (Peralta et al., 2020). *SFN* was reported to regulate cell cycle progression in bovine mastitis *via* genome-wide association (Miles et al., 2021). *IDH1* was identified as a candidate gene in the milk transcriptome of dairy cattle implicated in innate immunity by pathway and network analysis (Banos et al., 2017). *ABCG2* gene, which is regulated by the mammary gland, responsible for the active secretion of several compounds into milk (Otero et al., 2015).

The DT model identified the *LOC407171* gene as a critical player in mastitis disease in this study. *LOC407171* has been validated using an attribute weighting algorithm and a machine-learning algorithm. In addition, *SFN* and *IDH1* were identified using attribute weighting and machine-learning techniques, with *IDH1*, validated using WGCNA. Furthermore, *ABCG2* is recognized using weighted attributes, machine learning, and WGCNA. In addition, machine learning, attribute weighting, PPI network, and WGCNA were used to confirm *PTPRC*.

We examined possible changes in gene expression and connectivity during mastitis, and it was concluded that genes involved in the development, proliferation, and differentiation of cells in the mammary gland, as well as genes involved in immune system improvement, were primarily altered in their expression.

## Conclusion

Because of the complexity of mastitis disease in dairy animals, far more relevant research is required to identify biomarkers associated with mastitis. The current study's findings from meta-analysis, WGCNA, and machine-learning approach allow us to represent the primary contribution to our understanding of the most valuable genes for *E. coli* mastitis, which may provide a more robust biosignature and thus serve as reliable biomarker candidates in future studies. Our study suggests that all identified genes affect mastitis disease *via* their immune system–related functions.

## Data Availability Statement

The original contributions presented in the study are included in the article/Supplementary Material, further inquiries can be directed to the corresponding author.

## Author Contributions

NG: research concept and design, data analysis and interpretation, wrote the article, and final approval of the article. JS, SR, and KH: wrote the article. BP: data analysis, interpretation, wrote the article, and final approval of the article. All authors contributed to the article and approved the submitted version.

## Conflict of Interest

The authors declare that the research was conducted in the absence of any commercial or financial relationships that could be construed as a potential conflict of interest.

## Publisher's Note

All claims expressed in this article are solely those of the authors and do not necessarily represent those of their affiliated organizations, or those of the publisher, the editors and the reviewers. Any product that may be evaluated in this article, or claim that may be made by its manufacturer, is not guaranteed or endorsed by the publisher.
