# Supplementary material for: Integrative Systems Biology Analysis Elucidates Mastitis Disease Underlying Functional Modules in Dairy Cattle
Source: Front Genet. 2021 Oct 8;12:712306. doi: 10.3389/fgene.2021.712306 (PMC8531812; doi:10.3389/fgene.2021.712306)
Supplement: Supplementary file 6 [file Table_1.DOCX]

**Integrative Systems Biology Analysis Elucidates Mastitis Disease Underlying Functional Modules in Dairy Cattle**

**Nooshin Ghahramani ^1^*, Jalil Shodja^2^, Seyed A. Rafat^2^, Bahman Panahi^3^, Karim Hasanpur^2^**

^1-^ Department of Animal Science, Faculty of Agriculture, University of Tabriz, Tabriz, Iran

^2-^ Department of Animal Science, Faculty of Agriculture, University of Tabriz, Tabriz, Iran

^3 -^Department of Genomics, Branch for Northwest & West region, Agricultural Biotechnology Research Institute of Iran (ABRII), Agricultural Research, Education and Extension Organization (AREEO), Tabriz, Iran

***Corresponding author:**

Tel: +989911454745

Email: [gh.nooshin69@gmail.com](mailto:gh.nooshin69@gmail.com)

**Supplementary S1.** Detailed information of mapping rate of samples in bovine mastitis.

| Accession ID | Species | Run Accession | Read Count | Mapping Rate |
| --- | --- | --- | --- | --- |
| GSE75379 | Bos Taurus (Holstein) |  |  |  |
|  | | SRR2960020 | 46605503 | 87.6% |
|  |  | SRR2960023 | 32024574 | 78.6% |
|  |  | SRR2960026 | 58872438 | 81.8% |
|  |  | SRR2960029 | 25186805 | 96.7% |
|  |  | SRR2960032 | 25250142 | 90.3% |
|  |  | SRR2960035 | 33507969 | 95.4% |
|  | | SRR2960019 | 23208121 | 92.4% |
|  |  | SRR2960021 | 39639826 | 85.9% |
|  |  | SRR2960022 | 27786482 | 91.3% |
|  |  | SRR2960024 | 31267245 | 92.4% |
|  |  | SRR2960025 | 40815045 | 79.5% |
|  |  | SRR2960027 | 38433820 | 87.6% |
|  |  | SRR2960028 | 31515934 | 93.6% |
|  |  | SRR2960030 | 37946585 | 95.8% |
|  |  | SRR2960031 | 32578280 | 89% |
|  |  | SRR2960033 | 31322158 | 94.6% |
|  |  | SRR2960034 | 34722192 | 82% |
|  |  | SRR2960036 | 19791176 | 78.8% |

| Accession ID | Species | Run Accession | Read Count | mapping rate |
| --- | --- | --- | --- | --- |
| GSE159286 | Bos Taurus (Holstein) |  |  |  |
|  | | SRR12800530 | 13716160 | 92.4% |
|  |  | SRR12800531 | 15402434 | 79.5% |
|  |  | SRR12800532 | 19710523 | 87.6% |
|  |  | SRR12800533 | 13443600 | 93.6% |
|  |  | SRR12800534 | 16818381 | 95.8% |
|  |  | SRR12800535 | 20836200 | 89% |
|  |  | SRR12800536 | 15240224 | 94.6% |
|  |  | SRR12800537 | 17807570 | 82% |
|  |  | SRR12800538 | 16621830 | 78.8% |
|  |  | SRR12800539 | 20306251 | 92.4% |
|  | | SRR12800540 | 16483632 | 87.6% |
|  |  | SRR12800541 | 16476159 | 78.6% |
|  |  | SRR12800542 | 15620385 | 81.8% |
|  |  | SRR12800543 | 18289440 | 96.7% |
|  |  | SRR12800544 | 15543779 | 90.3% |
|  |  | SRR12800545 | 17620125 | 95.4% |
|  |  | SRR12800546 | 15579639 | 92.4% |
|  |  | SRR12800547 | 14872753 | 85.9% |
|  |  | SRR12800548 | 17420670 | 91.3% |
|  |  | SRR12800549 | 15168468 | 87% |
|  |  | SRR12800550 | 17120876 | 89% |
|  |  | SRR12800551 | 20383093 | 90% |
|  |  | SRR12800552 | 14354183 | 94% |
|  |  | SRR12800553 | 17553734 | 89% |
|  |  | SRR12800554 | 22038576 | 78% |
|  |  | SRR12800555 | 15956590 | 80% |
|  |  | SRR12800556 | 21699064 | 89% |
|  |  | SRR12800557 | 21439657 | 78% |
|  |  | SRR12800558 | 18436986 | 80% |
|  |  | SRR12800559 | 9058119 | 80% |
|  |  | SRR12800560 | 8028389 | 87% |
|  |  | SRR12800561 | 19274143 | 89% |
|  |  | SRR12800562 | 15859075 | 80% |
|  |  | SRR12800563 | 18985339 | 86% |
|  |  | SRR12800564 | 20806091 | 92.5% |
|  |  | SRR12800565 | 18547181 | 96% |
|  |  | SRR12800566 | 18173219 | 87% |
|  |  | SRR12800567 | 15247557 | 89% |
|  |  | SRR12800568 | 18281099 | 97% |
|  |  | SRR12800569 | 15333269 | 93% |
|  |  | SRR12800570 | 17863141 | 92.4% |
|  |  | SRR12800571 | 15222194 | 79.5% |
|  |  | SRR12800572 | 12281488 | 87.6% |
|  |  | SRR12800573 | 14172842 | 93.6% |
|  |  | SRR12800574 | 12587642 | 95.8% |
|  |  | SRR12800575 | 17514924 | 89% |
|  |  | SRR12800576 | 11538032 | 94.6% |
|  |  | SRR12800577 | 15032479 | 82% |
|  |  | SRR12800578 | 23681246 | 78.8% |
|  |  | SRR12800579 | 18581029 | 87.6% |
|  |  | SRR12800580 | 17114330 | 78.6% |
|  |  | SRR12800581 | 18553700 | 81.8% |
|  |  | SRR12800582 | 16312310 | 96.7% |
|  |  | SRR12800583 | 16710423 | 90.3% |
|  |  | SRR12800584 | 18415503 | 95.4% |
|  |  | SRR12800585 | 16632579 | 92.4% |
|  |  | SRR12800586 | 15026507 | 85.9% |
|  |  | SRR12800587 | 21314179 | 91.3% |
|  |  | SRR12800588 | 14364847 | 92% |
|  |  | SRR12800589 | 22127708 | 89% |
|  |  | SRR12800590 | 18111725 | 92.5% |
|  |  | SRR12800591 | 15986402 | 96% |
|  |  | SRR12800592 | 18768893 | 87% |
|  |  | SRR12800593 | 18199393 | 89% |
|  |  | SRR12800594 | 8894950 | 90% |
|  |  | SRR12800595 | 8563359 | 94% |
|  |  | SRR12800596 | 15354026 | 89% |
|  |  | SRR12800597 | 10916537 | 78% |
|  |  | SRR12800598 | 9075882 | 80% |
|  |  | SRR12800599 | 16729157 | 87.6% |
|  |  | SRR12800600 | 10133864 | 92.5% |
|  |  | SRR12800601 | 12363857 | 96% |
|  |  | SRR12800602 | 13246066 | 87% |
|  |  | SRR12800603 | 20749105 | 89% |
|  |  | SRR12800604 | 7113094 | 87.6% |
|  |  | SRR12800605 | 9681033 | 78.6% |
|  |  | SRR12800606 | 10040495 | 81.8% |
|  |  | SRR12800607 | 16028898 | 96.7% |
|  |  | SRR12800608 | 13810022 | 90.3% |
|  |  | SRR12800609 | 9848537 | 95.4% |
|  |  | SRR12800610 | 20258701 | 92.4% |
|  |  | SRR12800611 | 18243714 | 85.9% |
|  |  | SRR12800612 | 12160156 | 91.3% |
|  |  | SRR12800613 | 9739697 | 92% |
|  |  | SRR12800614 | 15009267 | 89% |
|  |  | SRR12800615 | 11418519 | 92.5% |
|  |  | SRR12800616 | 14790272 | 96% |
|  |  | SRR12800617 | 18198785 | 87% |
|  |  | SRR12800618 | 16818012 | 89% |
|  |  | SRR12800619 | 21284050 | 90% |
|  |  | SRR12800620 | 17094354 | 94% |
|  |  | SRR12800621 | 17848517 | 89% |
|  |  | SRR12800622 | 24291622 | 78% |
|  |  | SRR12800623 | 14063462 | 80% |
|  |  | SRR12800624 | 12755699 | 87.6% |
|  |  | SRR12800625 | 19675679 | 78.6% |
|  |  | SRR12800626 | 14615912 | 81.8% |
|  |  | SRR12800627 | 17894501 | 96.7% |
|  |  | SRR12800628 | 15405773 | 90.3% |
|  |  | SRR12800629 | 12659094 | 95.4% |
|  |  | SRR12800630 | 13009473 | 92.4% |
|  |  | SRR12800631 | 22369275 | 85.9% |
|  |  | SRR12800632 | 17337527 | 91.3% |
|  |  | SRR12800633 | 13400289 | 78% |
|  |  | SRR12800634 | 15241835 | 82% |
